# The Minimum Protein Staple? – Towards ‘bio’-Baldwin's rules *via* inter-phosphosite linking in the MEK1 activation loop†

**DOI:** 10.1039/d3sc04631a

**Published:** 2023-12-01

**Authors:** Sébastien R. G. Galan, Ritu Raj, Dimitrios Mamalis, Lyn H. Jones, Shabaz Mohammed, Benjamin G. Davis

**Affiliations:** a Department of Chemistry, University of Oxford Mansfield Road Oxford OX1 3TA UK Ben.Davis@rfi.ac.uk; b The Rosalind Franklin Institute Oxfordshire OX11 0FA UK; c Dana–Farber Cancer Institute Boston Massachusetts USA; d Department of Pharmacology, University of Oxford Mansfield Road Oxford OX1 3QT UK

## Abstract

In small molecule organic chemistry, the heuristic insight into ring-forming processes that was enabled by Baldwin's rules some 50 years ago proved a step-change in the role of mechanistically guided synthesis. It created a lens upon and marker of fundamental stereoelectronic and conformation-guided chemical processes. However, despite the widespread role of stereoelectronics and conformational control in Biology, no equivalent coherent exploitation of trapped, ring-forming processes yet exists in biomolecules. In the development of a minimal ring-closing process in intact proteins that might prove suitable in a coherent rule-set, we have tested *endo*-trig ring-closing conjugate thioether lanthionine (Lan) –CH_2_–S–CH_2_- formation as a limiting cyclization. Spontaneous Lan formation in proteins is rare if not non-existent and when found in natural product cyclic peptides it requires the mediation of corresponding biosynthetic enzymes as well as productive reactive conformations to guide it. Here, we show that within a conformationally flexible and functionally important protein loop – the MAPK kinase phosphorylation-targeted activation loop – Lan ring-closing is possible. Ring-closing proves to be critically dependent on the location of a *trig* electrophilic site in just one of two regioisomeric potential precursors to allow phosphosite-to-phosphosite ‘stapling’. This first example of spontaneous protein thioether ring-closing/‘stapling’ and its accessibility from just one precursor (despite the potential for both to form an identical ‘staple’) now reveals the potential for Lan formation not only as an accessible form of minimal stapling in proteins but also as an exquisitely sensitive probe of associated protein geometries. We suggest that the use of this (as well as the development of other such, intramolecular protein traps that are dependent on inherent protein-controlled reactivity rather than forced crosslinking) may allow the broader trapping and mapping of relevant, even minor, protein states. In this way, protein ring formation may enable a form of extended ‘bio-Baldwin's rules' that help to delineate relevant protein conformational space.

## Abstract 2

One-out-of-six regioselectivity allows the use of poly-Cys arrays to report on protein structure and reactivity in a biological extension of the Baldwin rules.

## Graphical abstract

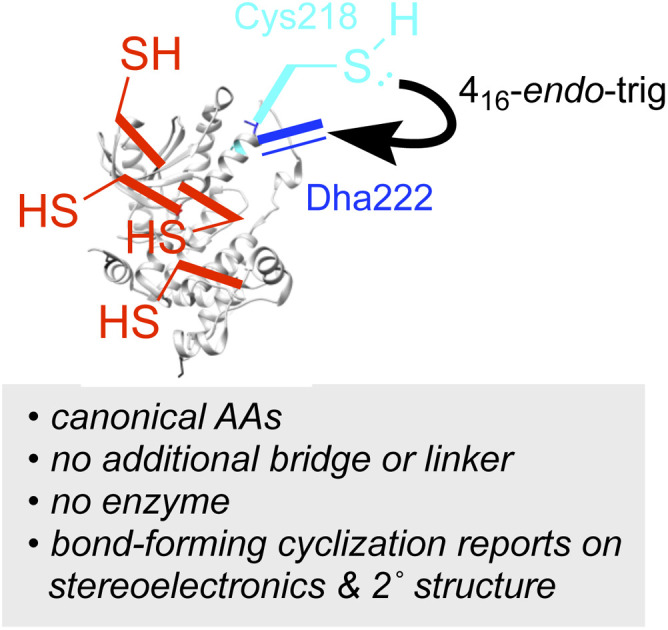

## Introduction

In the mid 1970s Jack Baldwin proposed a set of “rules” for predicting the cyclisation of small molecules.^1^ After a flurry of initial so-called anti-Baldwin examples from the community, a greater understanding was developed for the elegant underlying principles in these rules. At their heart was a central notion of stereoelectronic control, specifically the alignment of participating frontier orbitals and, in particular, consideration of cyclization *via* productive geometries that are coherent with archetypal principles in Organic Chemistry (*e.g.* Walden inversion,^2^ Bürgi-Dunitz angle^3^). This has eventually led over decades to deep insight into the conformational space of small molecules and how this may be used as a guiding principle in regio-, stereo- and chemo-selectivity.^4^

**Fig. 1:**
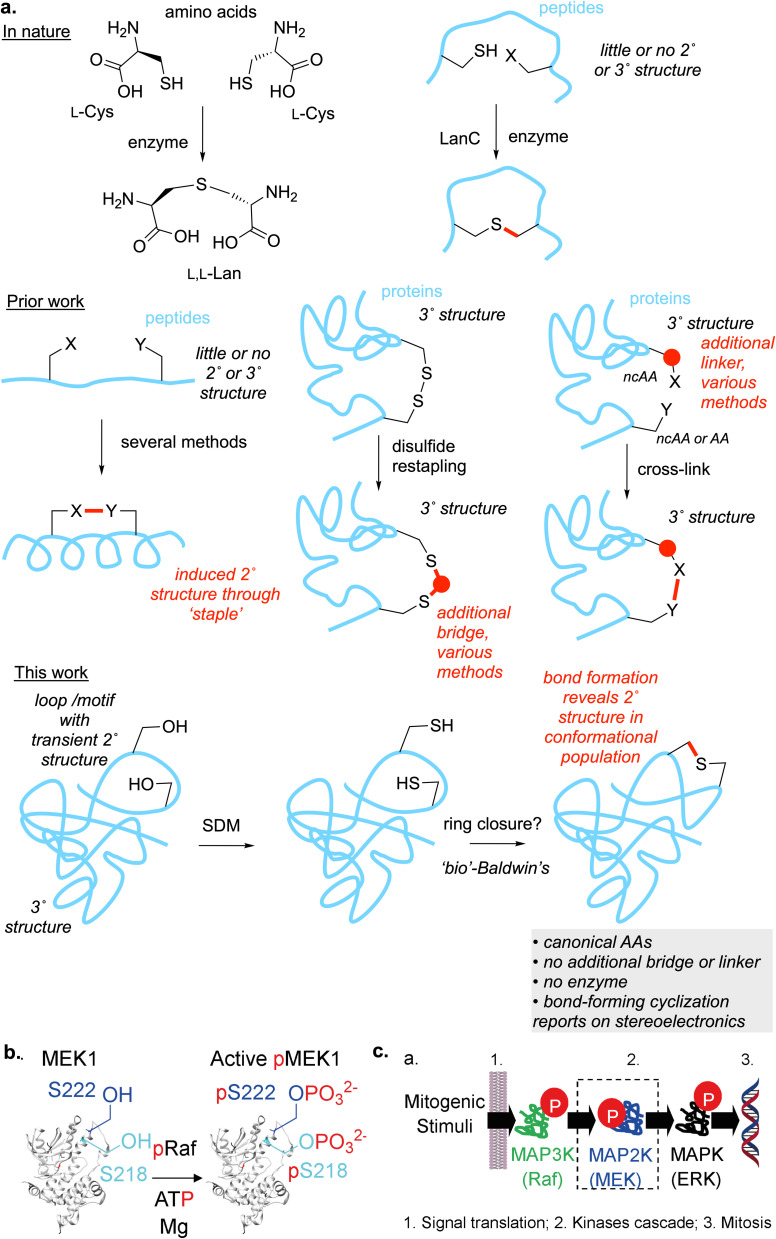
Stapling in MEK1. (a) (Top) In nature, Lan is formed enzymatically between amino acids and in peptides but not proteins. (Middle) Stapling methods used in peptides can stabilize extracted motifs and large cycles can bridge some proteins, albeit often with extended bridges or linkers and/or through use of reactive, non-canonical amino acids (ncAAs) that may not be driven by reactivity that reports on conformation; SDM = site directed mutagenesis. In this work: we propose Lan as a minimal staple that might, through a set of cyclization rules, report upon conformations that enable stapling chemistries, likely in minimal motifs more likely to be under conformational control of relevance to function. (b) Enzymatic phosphorylation at sites Ser218, Ser222 in its activation loop is the primary mode of activation of MEK1. (c) The Raf-MEK-ERK MAPK signaling cascade.

It may be argued that whilst stereoelectronic principles are prevalent in Biology,^5^ similar notions of cyclization events as a probe of underlying relevant conformers is still lacking. A systematic range of cyclization events that might report upon such conformational aspects would therefore be useful. However, to our knowledge, a register of such inherent, protein-controlled, reactivity events does not yet exist. Consequently, many (indeed most) known cyclization events are still limited to those that may be considered effectively to be macrocyclizations.^6^ These are therefore typically driven not by inherent conformational activation but instead largely by the exploitation of enhanced chemical reactivity (‘cross-linked’, Fig. 1a, middle right) within current strategies in Chemical Biology. This creates the risk of ‘forced’ events that do not allow fuller exploration of the population (including minor but reactive intermediates) of rapid Curtin–Hammett conformational pre-equilibria manifolds but that may instead report on reactivity that simply ‘out-strips’ and so traps the dominant (but perhaps less relevant) conformations. In addition, these macrocyclization events may also instead exploit broadscale surface affinity (*e.g.* hydrophobic, hydrogen-bonded or ligand-mimicry) effects where a reagent that is tethered *via* long (*e.g.* oligoethyleneglycol/(EG)_*n*_/‘PEG’) linker is essentially localized but mostly conformationally unconstrained. In this way such events do not readily report upon biologically-relevant conformations.

For example, many trapping events still exploit principles that are guided instead by mutual functional group reactivity (*e.g.* ‘click’-type reactions based upon azides, alkynes, strained unsaturation, and the interaction of sulfonyl fluorides^7–9^ with certain nucleophiles). It can be argued that this type of driven reactivity (albeit potentially kinetically latent to be enhanced with certain coupling partners) is less prone to the enhancement that might arise from correct *alignment* that can activate reactivity from essentially ‘zero background’ to a covalent event. We argue here that it is these conformationally- and stereoelectronically-enhanced events that are perhaps of more use given the greater selectivity that will eventually^10^ be needed in highly heterogenous, complex biological systems. Indeed, of late, it has been shown that reactive enhancements even allow simple alkyl halide protein sidechains to be used as accelerated *inter* molecular traps between proteins, when placed in the right sites within protein–protein interfaces,^11^ in a manner that complements the driven reactivity of other covalent protein traps.^12^ This highlights that new chemical functionality (or its rebirth^9^) is not always necessary to explore new modes of Chemical Biology. Here, we now explore initial strategies for generating minimally-sized *intra-*molecular traps that might serve as probes of the smaller ring-size end of a future spectrum of ring-cyclization rules.

## Results

### Design of a cyclizing reaction for testing ‘bio-Baldwin's’ rules

The activation loop of MAP(K) kinases is a primary example in nature of conformationally controlled protein function modulation.^13,14^ Human MEK1 (MAP2K1) is a dual-specific kinase in the Raf-MEK-ERK signaling pathway, centrally involved in key cell differentiation and proliferation pathways (Fig. 1c). In cells, phosphorylation of residues Ser218 and Ser222 in the loop (to phospho-Ser, pSer) by kinases activates MEK1 (Fig. 1b).^15–18^ ‘Active’ phosphorylated-MEK1 (pMEK1) then in turn phosphorylates ERK1, which activates the genetic machinery of mitosis. The activation of MEK1 has been directly linked to the conformational state of the activation loop and in particular the DFG motif (Asp208–Phe209–Gly210 in MEK1).^19^ It has been argued that the most commonly observed active conformations of the loop are found in so-called BLA_minus_ local minima (defined using XDF backbone dihedrals placed on Ramachandran plots at beta, left and alpha minima coupled with the side-chain DFG-Phe rotamer angle *χ*1 = −60°).^14^ Therefore, not only does the MEK1 activation loop provide an important test motif for exploration of conformational itineraries (as set out above), any strategies beyond observation of transience but perhaps, even to the locking or forcing of conformationally altered states in this loop, may be of direct catalytic and physiological relevance. We therefore chose to study this loop and these sites as substrates.

Whilst the intramolecular-linking/‘stapling’ of peptides through various methods has become a well-explored route for the locking of flexible loops in abstracted peptidic motifs that have been excised from proteins (Fig. 1a, middle left),^20^ the stapling of proteins however is more rare (Fig. 1a, middle middle).^6,21–23^ In these few cases, elegant use of non-canonical amino acids (ncAAs) has allowed modulation of protein thermostability through thioether formation between distinct protein domains over what might be formally considered to be large ring sizes (*e.g.* when counted *via* bonds ‘through’ the peptide backbone). Thus, so-called disulfide restapling^6^ has allowed Cys–Cys linkages to be alternatively bridged. Such methods have thus far been restricted to smaller proteins/large peptides. In all of these examples, whilst the use of computational prediction has been partially exploited^21^ it has also been highlighted^23^ that such methods, designed as they are only for the ground state precursors, may fail to account for likely transition state geometries in such stapling. Moreover, in the well-studied RiPP peptide biosynthetic pathways (where enzyme-catalyzed, and some spontaneous, thioether cyclization also occurs, Fig. 1a, top right) although some influence of peptide substrate primary sequence has been suggested as a method to train algorithmic (*e.g.* deep learning) methods it has also been concluded that there are, as yet, “*no easily identifiable predictive rules for all sequences*”.^24^

A minimal protein staple would be lanthionine (Lan, Fig. 1a, top left). This desulfurized –CH_2_–S–CH_2_- analogue of cystine –CH_2_–SS–CH_2_- could, in principle, be designed to create a close side-chain-to-side-chain interaction isostere for many residues (*e.g.* Ser-to-Ser –CH_2_–O–H⋯(H)O–CH_2_-, Ala-to-Ala *etc.* beyond cystine, Fig. 1a, bottom). It would also potentially not require additional bridging or the use of ncAAs in precursor proteins and so in principle could be chemically induced in native systems (even inside organisms). To our knowledge, whilst the lanthionine (Lan) linkage is quite widely found in ribosomally-derived natural products such as the lanthipeptide RiPPs,^25–27^ and is also rarely found in peptidoglycan peptide-to-peptide crosslinks (as a proposed DAP mimetic),^28^ the introduction of Lan in proteins is unknown. We therefore set out to develop a strategy for the introduction of a Lan-thioether staple that might mimic a possible transient Ser-to-Ser ‘bridge’ or interaction in the critical flexible activation loop of a MAP kinase and that would probe its associated conformations (Fig. 1a, bottom).

### Creation of putative cyclization substrate in MEK1 protein

**Fig. 2:**
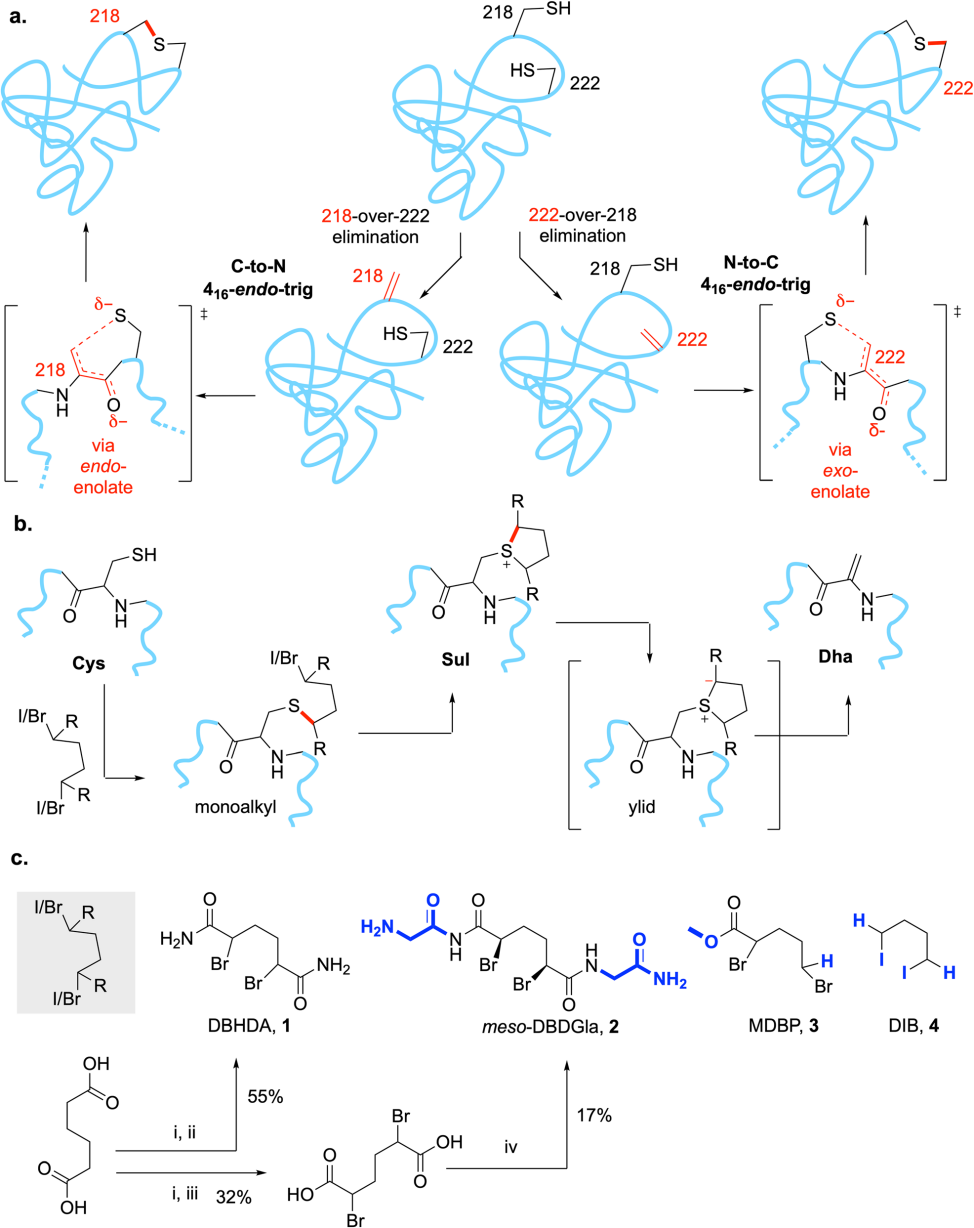
The Chemistry for a Minimal Staple in Proteins. (a) Regioselective differentiation in the formation of Dha from Cys would allow, in principle the creation of Lan from a dual Cys motif in MEK1. (b) Overall mechanistic sequence for site-selective incorporation of eliminated Dha residues (c) 1,4-Bis-alkylation/elimination agents. Synthesis: (i) SOCl_2_, reflux, 1.5 h then NBS, HBr/CCl_4_, reflux, 3 h; (ii) NH_4_OH, 0 °C, 1.5 h; (iii) H_2_O/THF (1 : 1), 0 °C, 2 h; (iv) SOCl_2_, reflux, 1.5 h then glycinamide then trituration in H_2_O.

The key Ser sites in MEK1 occur at Ser218 and Ser222 and are only 7–13 Å apart in observed structures. We reasoned that their proximity and possible intersite interaction could be probed by the creation of Lan as a minimal staple (see above) *via* a mutually-reactive electrophile·nucleophile pair. Whilst different pairs could be considered with varied geometries for ring closure,^1^ that of Cys as conjugate nucleophile coupled with dehydroalanine (Dha) as conjugate electrophile^29^ would allow testing of protein-relevant cyclization rules *via* an *endo*-trig pathway (Fig. 2a).

We have previously developed a 3-step, one-pot method (Fig. 2b) involving bis-alkylation/elimination that chemically converts free Cys residues through the use of 1,4-bis-halides, and, in particular, the reagent DBHDA 1 (Fig. 2c).^30^ Mechanistically these are thought to proceed through corresponding cyclic sulfoniums that, *via* intermediate ylids, allow mild E1cb-like elimination.^31^ In its typical, chemoselective, implementation in a protein substrate we would typically generate unique sites in a protein bearing free Cys for conversion to Dha (DBHDA is free Cys selective); this may be supplemented by the conversion, if benign, of untargeted Cys sites to near-isosteric, unreactive Ser.^29^ However, for two reasons we chose to adopt a more challenging dual regio- and chemo-selective strategy.

**Fig. 3:**
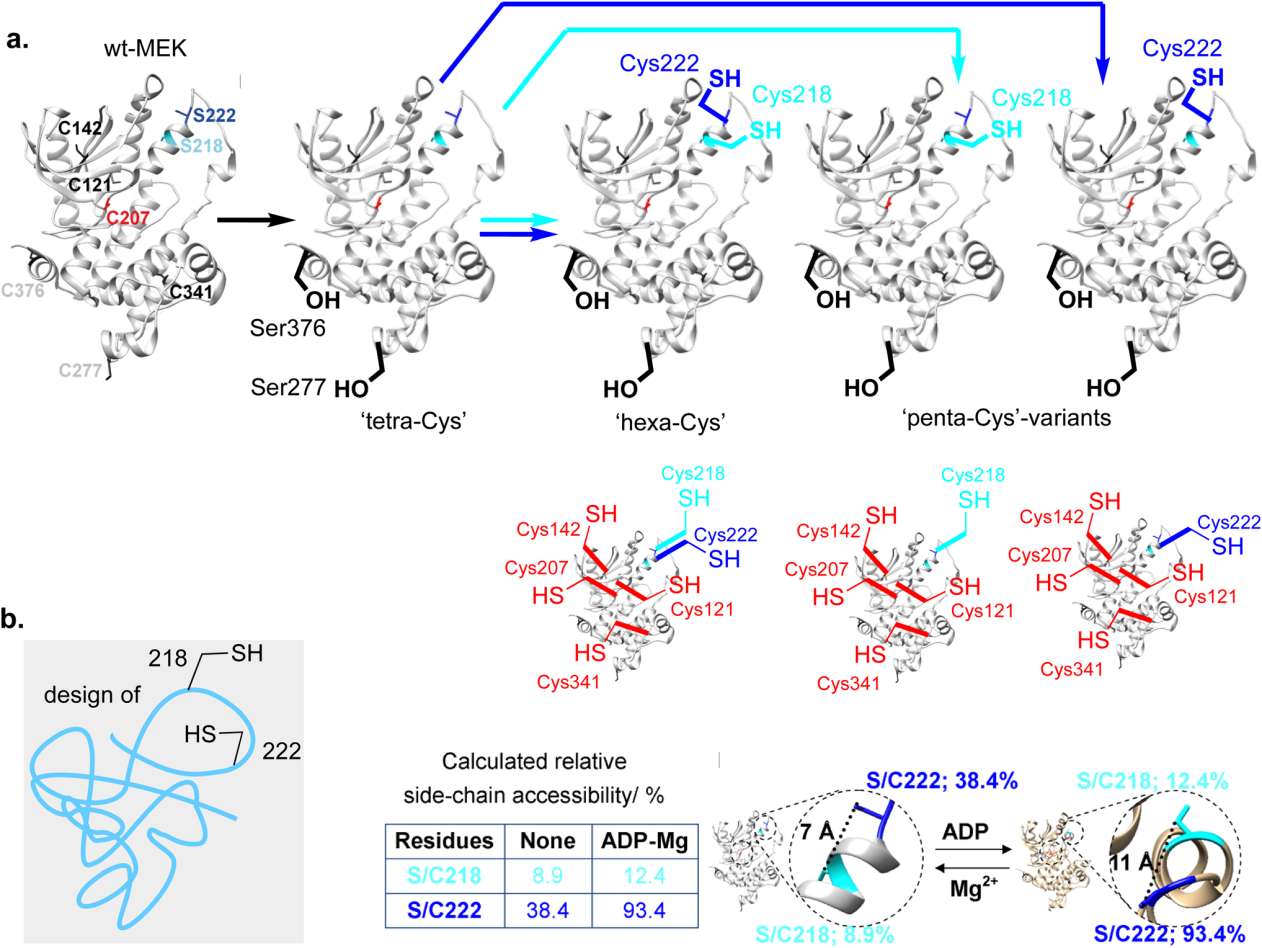
Design of a system in MEK1 for Lan stapling. (a) Site directed mutagenesis scheme to generate protein substrates in the design of a Lan staple. Wild type (wt) MEK1 (PDB 3W8Q) has six native free Cys residues (black, grey & red) and two activating Ser sites 218 (cyan) & 222 (blue). (b) Predicted side-chain reactive accessibility of sites 218 and 222 with and without ADP + Mg(ii).

First, MEK1 already contains six free, native Cys residues at positions 121, 142, 207, 277, 341 and 376 (Fig. 3a, left). Whilst mutation of Cys277 and Cys376 to Ser yielded a protein with essentially identical enzymatic properties to those of wt-MEK1,^32^ mutations of Cys121, Cys207 or Cys341 alter^33,34^ enzyme activity and Cys207 is a conserved residue that acts as target site for covalent inhibitors within the ATP-binding pocket;^35^ these observations indicated direct functional relevance necessitating their retention. We therefore chose the MEK1-Ser277–Ser376 double-point mutant as a ‘tetraCys’-background protein sequence within which we would need to avoid reaction of all four of the retained free Cys at Cys121, Cys142, Cys207, Cys341 (Fig. 3a).

Second, as a target substrate for cyclization we aimed to target a complementary pair of Dha and Cys at the sites in MEK1 (218 and 222) that are occupied by Ser218 and Ser222. Whilst, in principle, this could be achieved through access to Dha *via* other intermediate residues,^29^ we considered that we might implement an intriguing directly regioselective strategy from a dual free Cys precursor at these sites (*i.e.* Cys218 and Cys222) (Fig. 3a and b).

Together, for these reasons, we therefore chose the construction of a four-point ‘hexaCys’-MEK1 variant with six free Cys from the background MEK1-Ser277–Ser376 double-point mutant: MEK1-Ser277–Ser376–Cys218–Cys222 (Fig. 3a).

### ‘Reactive accessibility’ analysis suggests regioselective one-out-of-six Cys reaction in MEK1 substrate

Although challenging, starting from a dual Cys218 + Cys222 motif, selective formation of one Dha at either site in close proximity to an untouched Cys might allow creation of Lan thioether in MEK1 (Fig. 3). This would also need to be implemented without affecting non-target Cys (Cys121, Cys142, Cys207 and Cys341). Therefore, to accomplish this in ‘hexaCys’-MEK1 variant MEK1-Ser277–Ser376–Cys218–Cys222 would require ‘one-out-of-six’ differentiation of either Cys218 over Cys121, Cys142, Cys207, Cys222 and Cys341 or Cys222 over Cys121, Cys142, Cys207, Cys218 and Cys341.

Current methods using other chemistries for targeting one free Cys residue in proteins amongst other Cys are rare; they require engineering^36,37^ or privileged arrangements of residues that were not applicable in MEK1.^38^ In principle, however, the different environments of Cys should allow their direct chemical differentiation.^32,39^

We have previously demonstrated that a method of ‘reactive accessibility’ analysis^40^ allows useful prediction of the regioselectivity of chemistries in protein substrates containing multiply addressable functional groups, in that case multiple azides in a protein with multiple unnatural amino acid (uAA) azidohomoalanine (Aha) residues or multiple alkynes in multi-homopropargylglycine (Hpg) proteins. Encouraged also by prior utility in predicting ‘one-out-of-five’ and ‘two-out-of-six’ reactivity,^32^ we therefore set out to test the success of an essentially analogous ‘reactivity analysis’ process enabling instead our most testing case of regioselective ‘one-out-of-six’ Cys-elimination.

To discriminate potential reactivities, relative side-chain accessibility was estimated using a predictive computational approach^41^ based on accessible surfaces determined from corresponding X-ray crystal structures.^42^ This analysis applied to an unbound MEK1 structure [derived from PDB 3W8Q] predicted (Fig. 3b) that Cys might be added through site-directed gene mutagenesis (SDM) at sites 218 and/or 222 to give residues with useful predicted reactivity (as judged by % reactive accessibility) in the protein sequence background that we had designed (following the removal *via* Cys → Ser mutagenesis of relatively accessible residues Cys277 and Cys376 with 93.5, 21.9% accessibility respectively in MEK1-Ser277–Ser376 double-point mutant as a ‘tetraCys’-background); consistent with this, initial studies in the wt-MEK1 background gave intractable mixtures.

Importantly, the same predictive structural analysis of reactivity also suggested that in ternary complex MEK1·ADP·Mg [PDB 3EQI] accessibility to Cys207 is dramatically decreased (accessibility 22.4 → ∼0.1%), consistent with Cys207's location in the nucleotide binding site of MEK1. Notably, relative to a comparable binary complex MEK1·ATPγS [PDB 3W8Q], addition of Mg(ii) induces conformational change to give a ternary complex MEK1·ATPγS·Mg [PDB 3EQD] that usefully enhances accessibility of both sites (Cys222: 38.4 → 93.4%, Cys218: 8.9 → 15.2%, respectively, Fig. 3b). In this way, these analyses suggested that not only would reactivity in a ternary complex be enhanced at Cys222 (to 93.4% relative accessibility) but that this would lead to greater discrimination of Cys222 over Cys218 (from ∼4 : 1 Cys222 : Cys218 accessibility ratio to ∼8 : 1). These analyses prompted additional consideration of nucleotides^43,44^*i.e.* ADP and cofactors *i.e.* Mg(ii) as enhancing co-reagents to usefully modulate regioselectivity.

### Reagent variation creates a desulfurized protein variant consistent with regioselective one-out-of-six Cys reaction

In the 3-step conversion of Cys to Dha (Fig. 2b), the first irreversible alkylation step is rate- (and hence regio-selectivity) determining. We reasoned therefore that reagent tuning (without losing reactivity in steps 2–3) could therefore allow the control required to exploit the predictions of differential reactivity gained from structural ‘reactive accessibility’ analysis (see above).

We therefore chose four systematically-altered reagents^30,32,45^ (Fig. 2c) that would be expected to vary greatly in not only their reactivity in this first regioselectivity determining step but also their likely reactivity in later non-regioselectivity determining steps: DBHDA^30^1 and DBDGla^32^2 (both dual secondary bromide sites alpha to carboxyl with varied bulk), MDBP^45^3 (one secondary bromide site alpha to carboxyl plus one primary bromide) and DIB^30,32^4 (dual primary iodide sites). All retain the ability to create the 5-membered ring sulfonium intermediate that is critical to Dha formation^30^ yet are likely to differ in their ability (1, 2, 3 > 4) to sustain corresponding ylid intermediate^31^ and so likely to undergo elimination to Dha at differ rates.

**Fig. 4:**
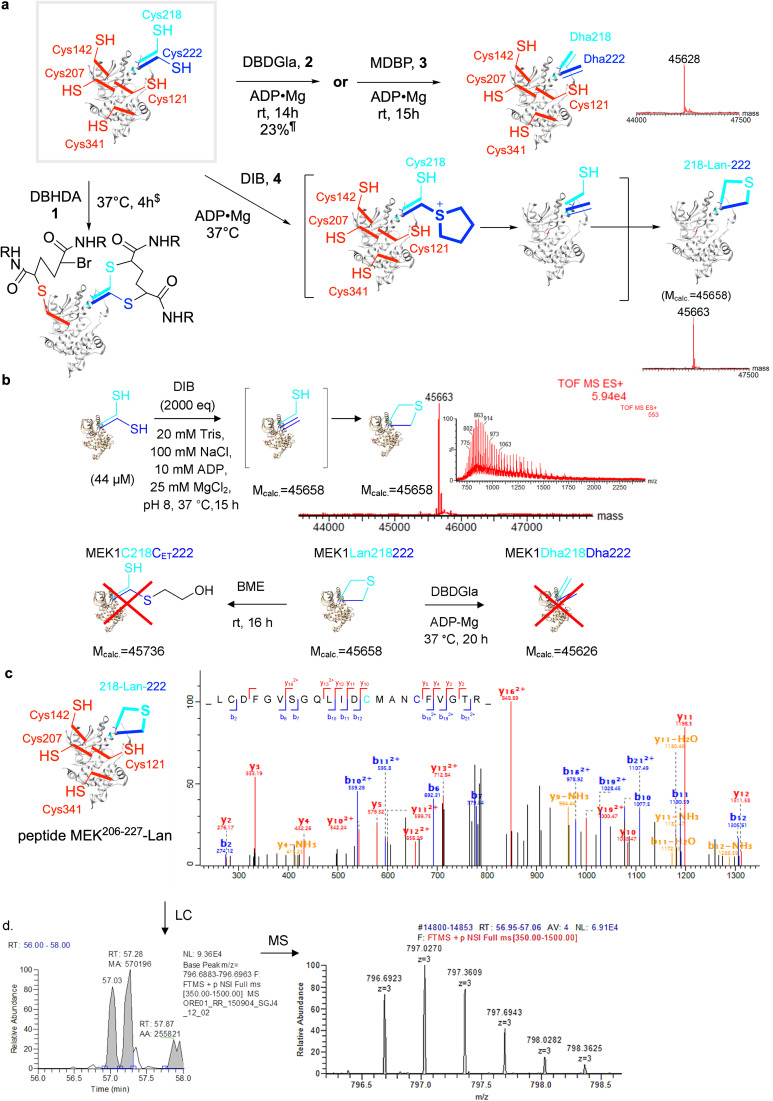
Creation of the first ‘lanthiprotein’ through minimal stapling. (a) Protein lanthionine formation from MEK1-C218C222 *via* regioselective generation of Dha222 and intramolecular reaction with nearby Cys218. Prior studies in MEK1 suggested^32^ a wide spectrum of reagent activities from the panel (Fig. 2c) that might allow differential installation of Dha.^$^*M*_obs._ = 46 057.^¶^*M*_obs._ = 45 627 (plus other side products, *M*_obs._ = 45 658, 18% plus 45 951, 59%). (b) Formation of lanthionine MEK1-Lan218222 from MEK1-Cys218–Cys222 with DIB *M*_calc._ = 45 658, *M*_obs._ = 45 663. Treatment of the product with β-mercaptoethanol (BME) and the addition of DBDGla suggested that neither Dha222 nor Cys218 were present in the mono-desulfurized product. (c) MSMS of tryptic digest peptides confirmed the formation of lanthionine; observation of Lan in MEK^206–227^ shown. (d) MS1 chromatogram suggested a DL/LL – Lan epimeric mixture.

We tested their reactivity against key substrate ‘hexaCys’-MEK1 variant with six free Cys from the background MEK1-Ser277–Ser376 double-point mutant: MEK1-Ser277–Ser376–Cys218–Cys222 (Fig. 4). This gave rise to strikingly different results. First, DBHDA 1 alone showed no clear Dha formation and only a product consistent with alkylation and no conversion to Dha (Fig. 4a); its less discriminate nature under these conditions led to it being discounted for further reactions in this system. Second, its bulkier close bis-amide analogue DBDGla 2 proved more productive, especially in the predicted beneficial presence of ligands, but nonetheless DBDGla 2 + ADP·Mg whilst successful in forming Dha did so with essentially no regioselectivity (dual at site 218 and 222) and then only modestly, giving only low conversion to MEK1-Dha218–Dha222 (23%, 14 h plus side products). DBDGla 2 is thus seemingly capable of regioselective reaction with Cys222 and Cys218 over other Cys but not in regioselectively discriminating Cys222 from Cys218. Next, MDBP 3 + ADP·Mg, whilst more reactive, also proved essentially non-regioselective cleanly giving MEK1-Dha218–Dha222 albeit in good conversion^32^ in 15 h (Fig. 4a). Finally, use of DIB + ADP·Mg whilst slower led to a seemingly clean mono-desulfurization product with intact protein LCMS indicating loss of one H_2_S equivalent (Fig. 4a).

### Characterization of regioselective protein product reveals a minimal Lan ‘staple’

This observed monodesulfurization driven by DIB could potentially have arisen through the formation of products *via* several modes; their products would be isobaric. These included: (i) non-regioselective mixed-site Dha formation; (ii) regioselective Dha formation (*e.g.* Cys222 over Cys218); alone; (iii) non-regioselective mixed site Dha formation and cyclization from all precursors; (iv) regioselective Dha formation followed by cyclization.

To distinguish (i)/(ii) from (iii)/(iv) we tested monodesulfurized-MEK1 product protein in two ways. First, for further alkylative reaction we used DBDGla 2 that is reactive with both free Cys222 and Cys218 but not other Cys in MEK1; no reaction was observed. Second, we tested for the presence of Dha in monodesulfurized-MEK1 through the addition of an excess of an external conjugate nucleophile, thiol β-mercaptoethanol; again, no reaction was observed. Together, these data revealed the absence of either free Cys or Dha residues at sites 218 and 222 in a monodesulfurized-MEK1 protein, consistent with putative formation of a cyclization product *via* Lan formation.

Next, the unambiguous formation of Lan between sites 218 and 222 was confirmed by LC-MSMS of peptide MEK^206–227^ derived from tryptic digest of monodesulfurized-MEK1 product protein (Fig. 4c). This form of ‘peptide mapping’ unequivocally confirmed the formation Lan218–CH_2_–S–CH_2_-222 as indicated by fragment analysis. Moreover, interestingly, the corresponding peptide chromatogram suggested the near equimolar formation of two isomers (∼44 : 56). We interpret this as arising from the formation an LL : LD diastereomeric peptide mixture that is epimeric at Cys222. In corresponding RiPP peptide cyclizations to Lan, those that proceed *via* C-to-N cyclization (*i.e.* arising from a Cys residue towards the C-terminus acting as a nucleophile) typically produce only one stereoisomer (*via* presumed *endo*-enolates, Fig. 2a) whilst those arising from N-to-C do not.^46^ The low stereoselectivity that we observe is therefore consistent with the presumed, designed N-to-C cyclization (218-to-222) *via* an *exo*-enolate^47^ arising from the regioselective generation by DIB in of Dha222 in the presence of Cys218. It also apparently discounts the formation of Lan *via* a direct stereoselective S_N_2 process *via* direct displacement of intermediate sulfonium that would have given only one LL peptide stereoisomer. Elegant studies delineating low intermolecular diastereoselectivity are also consistent with these observations.^48^ Together, we interpret these data as being most consistent with designed mode (iv) *via* formation of Dha222 followed by attack of Cys218 *via* an *endo*-trig cyclization pathway.

### Single Cys variants in the 218 + 222 motif are consistent with a mechanism of cyclization *via* regioselective Cys222 over Cys218 ‘one-out-of-six’ differentiation

These striking observations of the formation of a first protein Lan ‘staple’ were consistent with our designed strategy of regioselective differentiation (mode (iv)). However, we cannot fully discount contributions from mode (iii) with no underlying regioselective discrimination *i.e.* arising from mixed Dha222 + Cys218 plus Cys222 + Dha218 with both intermediates cyclizing to the observed Lan218–CH_2_–S–CH_2_-222 product. To test these possibilities further we used corresponding single Cys variants^32^ in the 218 + 222 motif (*i.e.* ‘pentaCys’ regioisomeric at sites 218 and 222) to probe their differing reactivities with DIB.

**Fig. 5:**
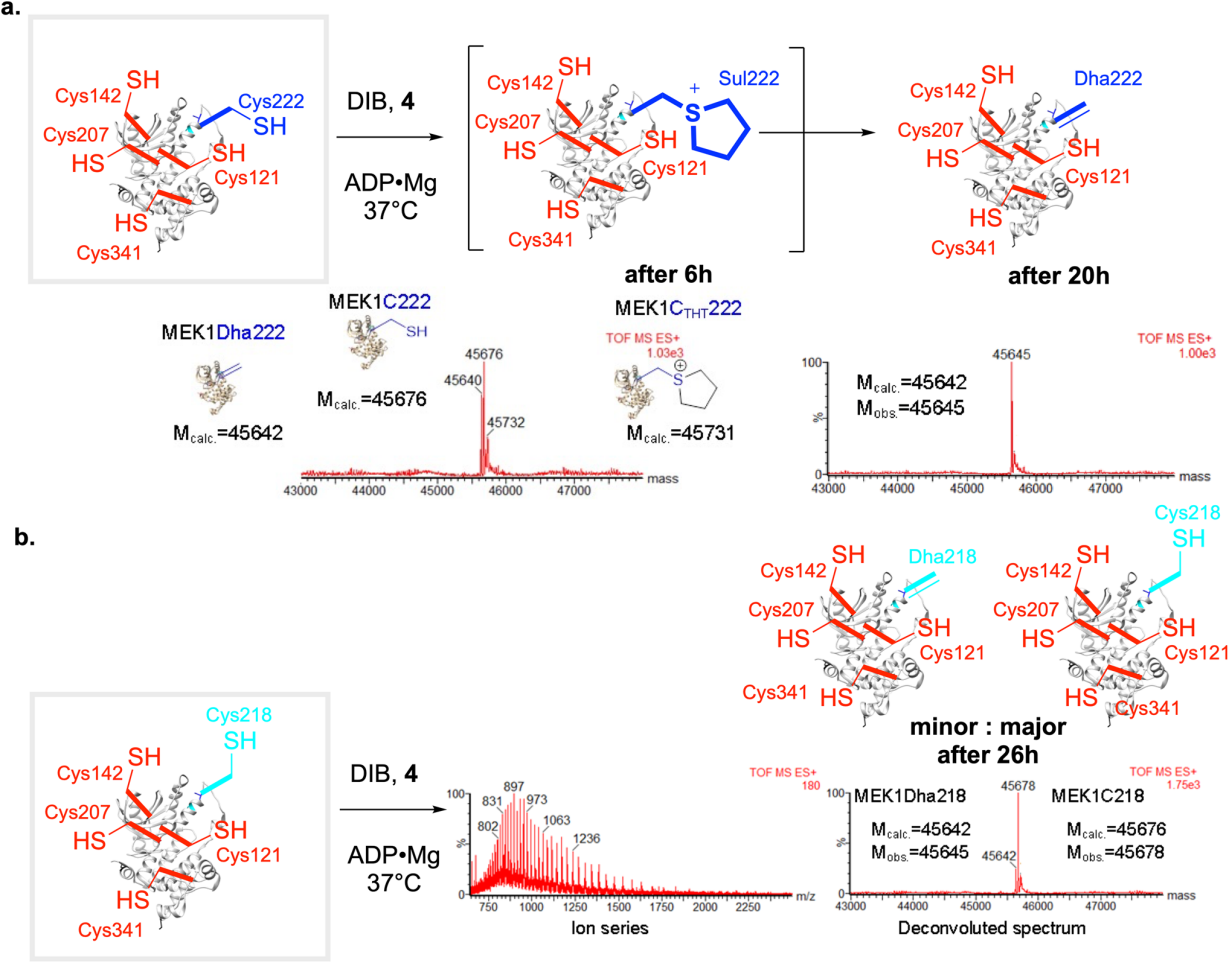
Penta-Cys models support strong regioselectivity by DIB in the formation of the Lan staple. (a) For penta-Cys MEK-Cys222 reaction with DIB was monitored using LCMS-ESI-TOF and after 6 hour showed a mixture MEK1-Dha222 (*M*_obs._ = 45 640, *M*_calc._ = 45 642), MEK1C222 (*M*_obs._ = 45 676, *M*_calc._ = 45 676) and the sulfonium intermediate MEK1-Sul222 (MEK1C_THT_, *M*_obs._ = 45 732, *M*_calc._ = 45 731). The formation of the first alkylation intermediate MEK1C_IB_222 (*M*_calc._ = 45 858) was not observed. Full conversion to MEK1Dha222 was observed after 20 hours. (b) By contrast for regioisomeric penta-Cys MEK-Cys218 only ∼15% conversion of MEK1-C218 (*M*_obs._ = 45 678, *M*_calc._ = 45 676) to MEK1-Dha218 (*M*_obs._ = 45 642, *M*_calc._ = 45 642) was observed after 26 hours.

Reaction of ‘pentaCys’-MEK1-Ser277–Ser376–Cys222 with DIB under essentially identical conditions used for the ‘hexaCys’ system (+ADP·Mg) formed the corresponding MEK1-Dha222 product with full conversion from MEK1-C222 in 20 h. The ‘one-out-of-five’ regioselectivity of reaction at Cys222 over other background Cys in MEK in this ‘pentaCys’ protein construct was again confirmed by LC-MSMS (Fig. 5a). Notably, consistent with reagent modulation (see above), initial bis-alkylation was rapid to form a sulfonium ion that was slowly reactive yet interestingly stable enough to be directly visible by intact protein LC-MS after 6 h (Fig. 5a). Such visibility of intermediate sulfoniums by LC-MS is rare,here starts to set the groundwork,^31^ as they usually eliminate spontaneously. This observed slower elimination for DIB in comparison with other alkylation agents (DBHDA, DBDGla, MDBP) is consistent with a lower ability of the corresponding unsubstituted sulfonium to support the intermediate ylid implicated in E1cb-type elimination.^31^ In addition, initial mono-alkylation by DIB was not visible by LCMS, as can be observed with other alkylating agents (DBHDA, DBDGla, MDBP); this is again consistent with reagent design.

Strikingly, reaction of regioisomeric ‘pentaCys’-variant MEK1-Ser277–Ser376–Cys218 under essentially identical conditions led to negligible MEK1-Dha218 formation (∼15% even after 26 h, Fig. 5b). Together these data are consistent with a mode (iv) cyclization *via* regioselective Cys222 over Cys218 ‘one-out-of-six’ differentiation *via* a Dha222 + Cys218 pair. To our knowledge, this use of chemo-plus regio-selective control to drive ‘stapling’ cyclization represents a unique mode of conformational control in proteins.

## Conclusions

We describe here the first examples of what might be considered to be a minimal protein staple through the formation of a lanthionine (Lan) thioether. We designed and implemented a process of sequential regioselective generation of a conjugate electrophile (Dha) in the presence of a nearby pendant conjugate nucleophile (Cys) for the direct formation of this staple. This necessitated a ‘one-out-of-six-Cys’ regioselective strategy based upon ‘reactive accessibility’ analysis and that also appears to have been facilitated by the use of a reagent that displays lower overall bis-alkylation–elimination kinetics in the formation of Dha, DIB.

This conjugate electrophile-plus-conjugate nucleophile process parallels those catalyzed enzymatically in RiPP lanthi-peptide biosynthesis for the formation of cyclic structures.^25–27^ Here, we have now used an analogous chemical process that has allowed expansion to the first example of a ‘lanthi-protein’.

We propose that these types of ‘cyclization for stapling’ methods might start to enable the analysis of conformational populations within proteins through the development of a set of protein rules for ring-closure.^1^ For this reason we chose a semi-mobile loop region that has important conformational consequences in the activation loop of MEK1; the location of site 218 in an α-helix (222 is in a flexible loop) is also correlated with its more marked effect upon activation *via* mono-phosphorylation. Notably the conjugate electrophile–nucleophile chemistry that we use brings together two residues which are relatively close in space but not close enough to form a spontaneous intramolecular disulfide. Therefore successful covalent cyclization reaction in a system that is governed by an *endo*-trig cyclisation suggests that stereoelectronic control coupled with essential irreversibility is here important. This pertinently highlights that not all cyclization chemistries are the same. Whilst we have no direct evidence to support this, the separation here of reacting residues by an *i*+4 distance immediately suggests speculation on the possible stabilisation of an helical interaction.

In turn, this gives initial promising evidence that different cyclization chemistries may report on different conformers in a manner that we suggest mimics (albeit poorly) the rules of Baldwin for small molecules. Whilst it is obvious that many more examples will be required before this is fully tested, the initial example we reveal here starts to set the groundwork. Other possible reaction types of clear interest include protein reactions that involve the intermediacy of free-radicals^49^ (*e.g.* thiyl-ene C–S-bond formation *via* S˙,^50^ or desulfurative C–C-bond formation *via* C˙,^51^) that will likely test the role of SOMO *vs.* HOMO alignment. We now propose a nomenclature for describing such intramolecular cyclizations that might be appropriate to proteins. This is a hybrid between the nomenclature used for intra-helical interactions developed by Bragg, Kendrew and Perutz^52^ with that proposed by Baldwin^1^ that utilizes descriptors of both residue-to-residue measures as well as ring-size. In this nomenclature [residue spacing_ringsize_-bond movement-hybridization] the Cys218-onto-Dha222 Lan formation that we observe here is a 4_16_-*endo*-trig cyclization. Our initial studies suggest here that a 4_16_-*endo*-trig cyclization C–S cyclization is favoured in this MEK1 motif over corresponding 4_17_-*exo*-tet S–S disulfide cyclizations. These observations further suggest that future studies should be aimed towards minimal staples with the potential to trap useful and relevant intra-protein interactions in a background of ‘null reactivity’ rather than use of larger-ring cyclization methods driven by reactivity.

If we are correct in our hypothesis that cyclizations may mimic or ‘observe’ transient protein conformations, the Lan system also offers the additional intriguing potential for stereochemical reporting. Here we already interpret our results for the apparent formation of two Lan DL/LL diastereomers as being consistent with an extension of the explanation proposed by van der Donk and coworkers for the governance by *endo vs. exo* enolates upon configuration found in peptides now also to proteins. It would be immediately intriguing to observe the converse cyclization of Cys222 onto a Dha218 that might generate a more-rigid, intramolecularly hydrogen-bonded (and so facially-differentiated) *endo*-enolate for protonation as has been suggested as a source of Lan stereoinduction;^47^ whilst the current methodology does not yet allow access to this pathway, experiments are in progress in our lab to find an alternative method. Whilst we interpret the isolation^32^ of stable Dha218 and Dha222 variants that bear Cys207 (and do not spontaneously cyclize) as being indicative of non-reactivity of Cys207 in forming Lan to these sites, it would also be of interest to explore alternative modes of cyclization (*e.g.* Cys218 or Cys222 onto Dha207) within MEK1. Furthermore, the generation of other conjugate electrophiles such as dehydrobutyrines (Dhbs) would enable further (*re vs. si*-face) stereoinduction that would extend the information that would be gained from the configuration of ‘stapled’ products.

Finally, It should be noted that the generation of a Lan staple from canonical, native amino acid residues would, in principle, allow its implementation (*via* appropriate elimination chemistry) in endogenously-expressed designed protein substrates without the need for unnatural amino acid residues, additional linkers or enzymatic post-translational activities. This suggests that such protein ring-cyclization rules as potential reporters of conformation, may in the future, be applicable even to more complex biological media as a means of trapping relevant confirmations (and their ‘read out’ using *e.g.* MSMS as we show here), perhaps even in living systems.

## Methods

### Reaction of Penta-Cys MEK1-Cys222 with DIB in the presence of ADP-Mg

MEK1-Cys222 (44 μM, 2.2 mg mL^−1^) in 20 mM tris, 100 mM NaCl, 10 mM ADP, 25 mM MgCl_2_, pH 8 buffer was treated with 1,4-diiodobutane, DIB (2000 eq. added as such). DIB is not fully miscible in aqueous buffer; efficient shaking is essential for reaction to occur. The mixture was shaken at 1000 rpm at 37 °C. The reaction was monitored using LCMS-ESI-TOF. LCMS after 6 hour showed a mixture MEK1-Dha222 (*M*_obs._ = 45 640, *M*_calc._ = 45 642), MEK1-Cys222 (*M*_obs._ = 45 676, *M*_calc._ = 45 676) and the sulfonium intermediate MEK1C_THT_ (*M*_obs._ = 45 732, *M*_calc._ = 45 731) (see ESI Fig. S1†).

The formation of the first alkylation intermediate MEK1C_IB_222 (*M*_calc._ = 45 858) was not observed. Full conversion to MEK1Dha222 was observed after 20 hours (see ESI Fig. S2†).

One aliquot of the reaction mixture was digested with trypsin using in-gel protocol. The digested peptides were analysed using the orbitrap method and processed by Peaks 7.0. LCMS-MS analysis of peptide 206–227 containing the residue 207 and 222 confirmed the position of Dha formation at position 222 (see ESI Fig. S3†).

### Reaction of penta-Cys MEK1-Cys218 with DIB in the presence of ADP-Mg

MEK1-Cys218 (44 μM, 22 mg mL^−1^) in 20 mM tris, 100 mM NaCl, 10 mM ADP, 25 mM MgCl_2_, pH 8 buffer was treated with 1,4-diiodobutane, DIB (2000 eq. added as such). The mixture was shaken at 1000 rpm at 37 °C. The reaction was monitored using LCMS-ESI-TOF. ∼15% conversion of MEK1-C218 (*M*_obs._ = 45 678, *M*_calc._ = 45 676) to MEK1-Dha218 (*M*_obs._ = 45 642, *M*_calc._ = 45 642) was observed after 26 hours (see ESI Fig. S4†).

### Reaction of hexa-Cys MEK1-Cys218–Cys222 with DIB in the presence of ADP-Mg

MEK1C218C222 (44 μM, 2 mg mL^−1^) in 20 mM tris, 100 mM NaCl, 10 mM ADP, 25 mM MgCl_2_ pH 8 was treated with 1,4-diiodobutane, DIB (2000 eq. added as such). The mixture was shaken at 600 rpm at 37 °C. The reaction was monitored using LCMS-ESI-TOF. Full conversion to MEK1-Lan218222 (*M*_obs._ = 46 663, *M*_calc._ = 46 658) after 15 hours (see ESI Fig. S5†).

The mass did not change upon the addition of an excess of either β-mercaptoethanol or DBDGla (12 mM final concentration). One aliquot of the reaction mixture was digested with trypsin using in-solution protocol. The digested peptides were analysed using the orbitrap method and processed by Maxquant. LCMS-MS analysis of peptide 206–227 containing the residue 207, 218 and 222 confirmed the formation of a thioether bond between both positions 218 and 222 (see ESI Fig. S6†).

Analysis of the LC trace of the LCMS-MS analysis showed two peaks for the desired mass corresponding to a mixture of epimers (see ESI Fig. S7†).

## Data availability

Please see attached ESI data file.†

## Author contributions

S. R. G. G., S. M. and B. G. D. designed the study. S. R. G. G., R. R. and D. M. conducted experiments. R. R. and S. M. conducted proteomic experiments and analyses. All authors analysed and interpreted data. B. G. D. wrote the manuscript. All authors read and commented on the manuscript.

## Conflicts of interest

There are no relevant conflicts arising from this study to declare.

## Supplementary Material

SC-015-D3SC04631A-s001
