# Supplementary material for: The Minimum Protein Staple? – Towards ‘bio’-Baldwin's rules via inter-phosphosite linking in the MEK1 activation loop
Source: Chem Sci. 2023 Dec 1;15(4):1306–17. doi: 10.1039/d3sc04631a (PMC10806838; doi:10.1039/d3sc04631a)
Supplement: SC-015-D3SC04631A-s001 [file SC-015-D3SC04631A-s001.pdf]

Electronic Supplementary Material (ESI) for Chemical Science.
This journal is © The Royal Society of Chemistry 2023

The Minimum Protein Staple? – Towards 'bio'-Baldwin's Rules via Inter-phosphosite Linking in the MEK1 Activation Loop

SUPPLEMENTARY DATA

Supplementary Figures

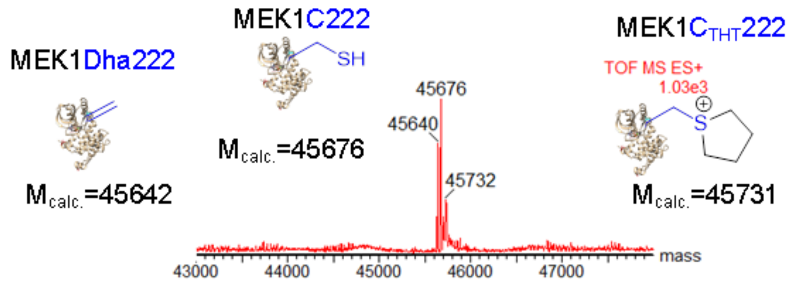

Supplementary Figure S1. Reaction of Penta-Cys MEK1-Cys222 with DIB in the presence of ADP-Mg after 6h.

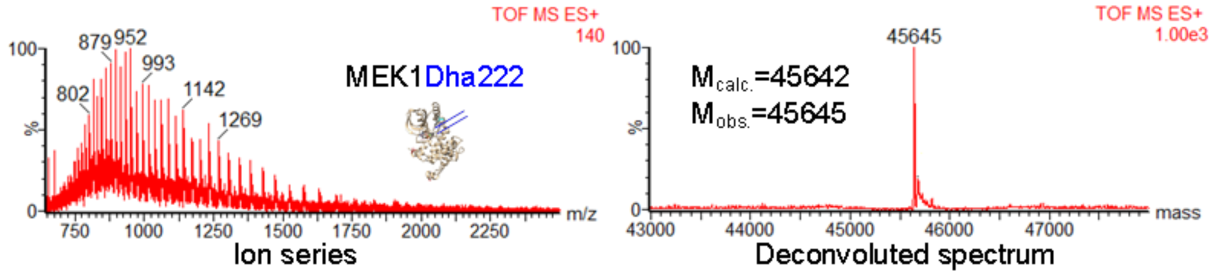

Supplementary Figure S2. Reaction of Penta-Cys MEK1-Cys222 with DIB in the presence of ADP-Mg after 20h.

Peptide	RT/min	m/z (charge)	ppm	Modifications	-10lgP	TIC	Max Intensity
206-227	37.74	1225.5529 (2)	-6.7	C_{ET}222, oxM219, C_{CAM}207	98.92	1.21E4	7.74E2

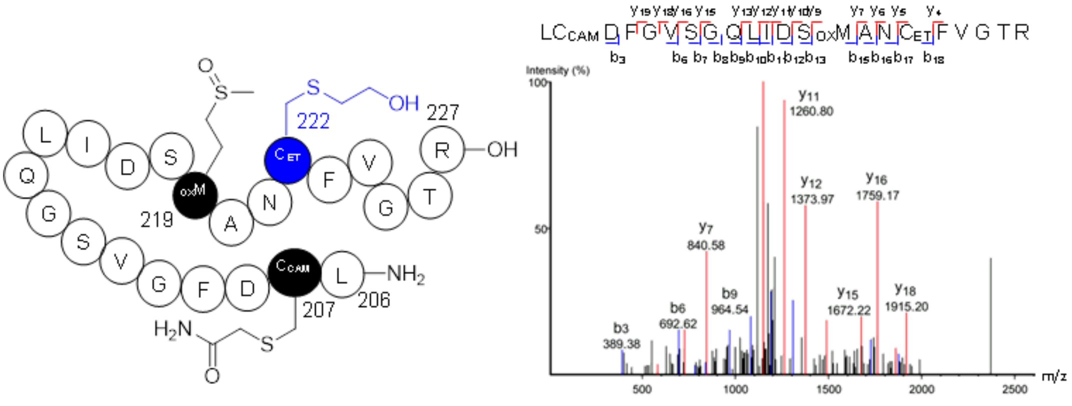

Supplementary Figure S3. MSMS Analysis of Product of Reaction of Penta-Cys MEK1-Cys222 with DIB in the presence of ADP-Mg.

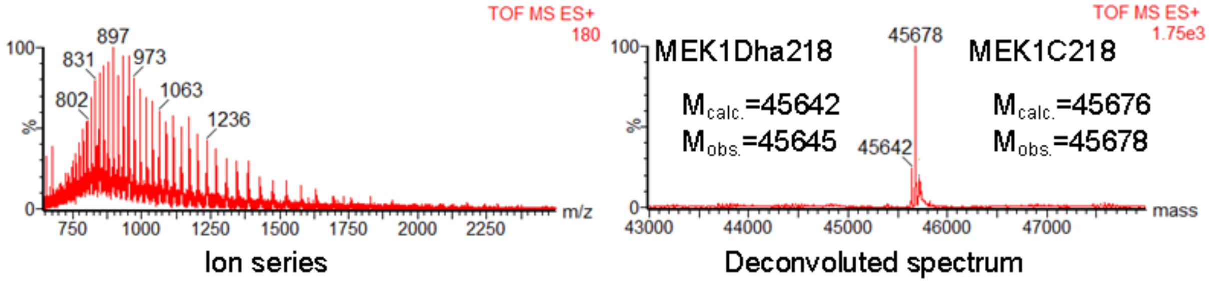

Supplementary Figure S4. Reaction of Penta-Cys MEK1-Cys218 with DIB in the presence of ADP-Mg after 26h.

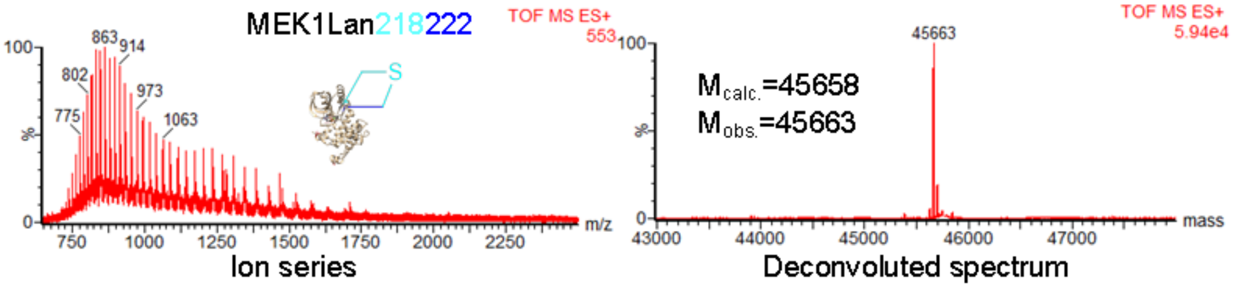

Supplementary Figure S5. Reaction of Hexa-Cys MEK1-Cys218-Cys222 with DIB in the presence of ADP-Mg after 15h.

Peptide	Mass (Da)	Score	Modifications	Method
206-227	2348.04	101	Lan218222, oxM219	ITMS CID

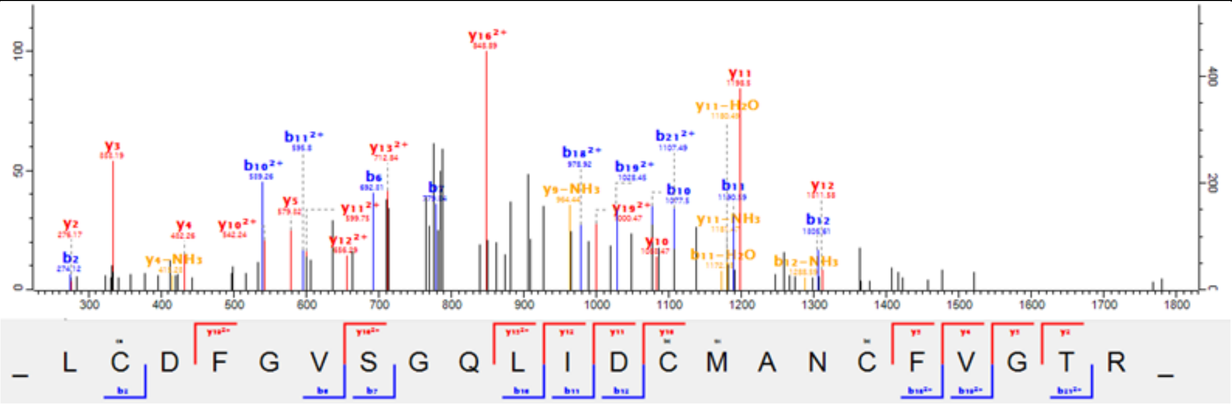

Supplementary Figure S6. MSMS Analysis of Product of Reaction of Hexa-Cys MEK1-Cys218-Cys222 with DIB in the presence of ADP-Mg.

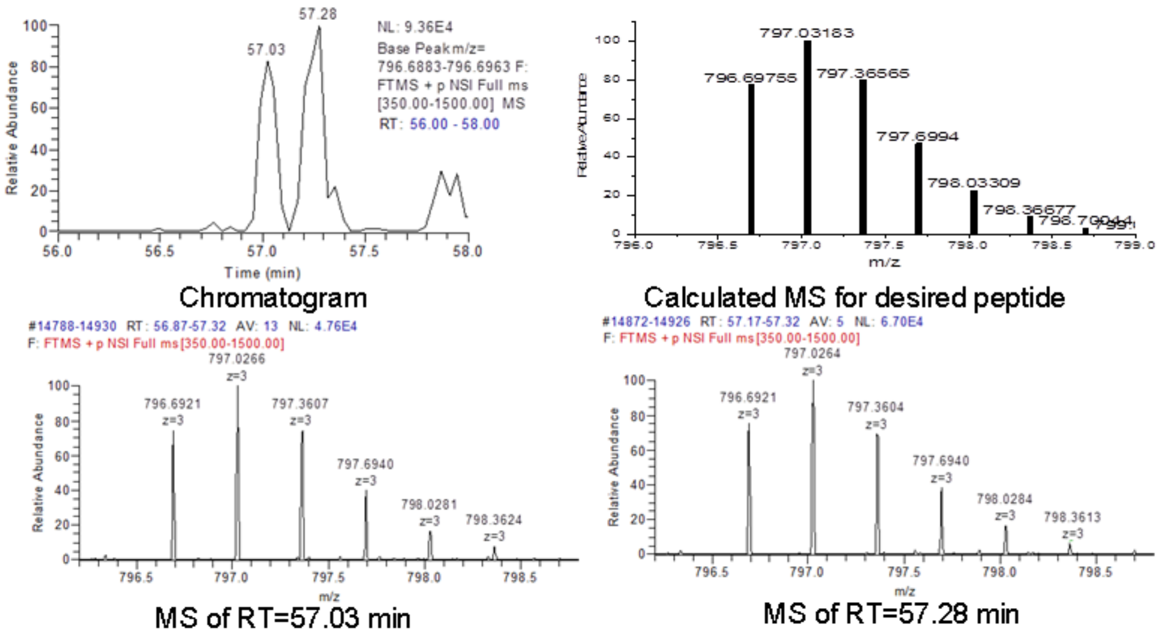

Supplementary Figure S7. LC trace of the LCMS-MS analysis of Reaction of Hexa-Cys MEK1-Cys218-Cys222 with DIB in the presence of ADP-Mg.
